# The complete chloroplast genome sequences of *Larix kaempferi* and *Larix olgensis* var. koreana (Pinaceae)

**DOI:** 10.1080/23802359.2017.1419092

**Published:** 2017-12-21

**Authors:** Sang-Chul Kim, Jei-Wan Lee, Min-Woo Lee, Seung-Hoon Baek, Kyung-Nak Hong

**Affiliations:** Division of Forest Genetic Resources, National Institute of Forest Science, Suwon, Republic of Korea

**Keywords:** *Larix kaempferi*, *Larix olgensis* var. *koreana*, complete chloroplast genome, phylogenetic analysis; Pinaceae

## Abstract

The complete chloroplast (cp) genome sequence of *Larix kaempferi* and *L. olgensis var. koreana* were determined by Ion torrent Platform sequencing in this study. The *L. kaempferi* cp genome was 122,158bp consists of two inverted repeat (IR) regions of 436bp each, a large single-copy (LSC) region of 65,394bp, and a small single-copy (SSC) region of 55,892bp. The chloroplast genome sequence of *L. olgensis* var. *koreana* was 122,573bp in length, consisting of two IRs (436bp), one LSC (65,597bp), and one SSC (56,104bp), and is longer than that of *L. kaempferi*. The genome contained 110 genes, including 71 protein-coding genes, 35 tRNA genes, and 4 ribosomal RNA genes. The 13 genes contain introns, including 12 genes with a single intron each and *ycf3* gene with two introns. And, the rps12 gene is a trans-spliced gene. The phylogenetic analysis revealed that all sampled species in Pinaceae formed a monophyletic clade with high bootstrap value. The genus *Larix* is closely related to *Pseudotsuga*.

The *Larix*, a prominent component of the boreal, montane, and subalpine forests, is widely distributed across North America, Asia, and Europe (Wei and Wang 2003). *Larix kaempferi* (Lamb.) Carrière was an afforested species introduced in Japan in 1910s; it was the second afforestation tree species after *Pinus densiflora* in Korea (Kang et al. 2016). In the present study, we assemble and characterize the complete chloroplast genome of *L. kaempferi* and the chloroplast of a native species, *L. olgensis* var. *koreana* (Nakai) Nakai based on next-generation sequencing. The annotated cpDNA of *L. kaempferi* and *L. olgensis* var. *koreana* has been deposited in the GenBank with the accession number MF990369 and MF990370.

The samples were collected from the conservation area of the Forest Genetic Resources Department of the National Institute of Forest Science (in Suwon) and its genomic DNAs were isolated from fresh leaves using a Plasmid SV mini kit (GeneAll, Korea) and stored in a DNA bank in the Forest Genetic Resources Department (NIFS_0122059344, 012059345). The whole genome sequencing was conducted on the Ion torrent Platform (Life Technologies, Carlsbad, CA). The sequenced fragments were assembled using Geneious 10.2.3 (Biomatters, Auckland, New Zealand; Kearse et al. 2012) with reference sequence of *L. decidua* (GenBank: NC016058). The gene annotations were performed using the Basic Local Alignment Search Tool (BLAST, BLASTX) available on the website of the National Center for Biotechnology Information. All the tRNA sequences were confirmed using the web-based online tool, tRNAScan-SE (Schattner et al. 2005) with default settings to corroborate tRNA boundaries identified by Geneious. The genome maps were generated using OGDraw (OrganellarGenomeDRAW; Lohse et al. 2007). The maximum likelihood (ML) tree searches and ML bootstrap searches were performed using the RAxML Blackbox web-server (http://phylobench.vital-it.ch/raxml-bb/, Stamatakis et al. 2008). The RAxML analyses were run with a rapid Bootstrap analysis using a random starting tree and 100 ML bootstrap replicates.

*Larix kaempferi*, the circular double-stranded cpDNA sequence of 122,158bp consists of two inverted repeat (IR) regions of 436 bp each, a large single-copy (LSC) region of 65,394bp and a small single-copy (SSC) region of 55,892bp, and *L. olgensis* var. *koreana* was 122,573bp in length, consisting of two IRs (436bp), one LSC (65,597bp), and one SSC (56,104bp), and is longer than that of *L. kaempferi*. The genome contained 110 unique genes, with additional copies of the *trnS*-GCU and *trnT*-GGU gene in the LSC region and only *trnI*-CAU gene in the IR region. In the whole chloroplast genome, 13 genes contain introns, including 12 genes with a single intron each and *ycf3* gene with two introns. The *rps12* gene is a trans-spliced gene with two duplicated 3′ end exons in IR regions and one 5′ end exon in the LSC region.

The phylogenetic analysis based on 63 protein-coding genes in 19 chloroplast genomes infer phylogenetic relationships among the main representatives of Pinaceae, and outgroups (Cupressaceae; Figure 1). The phylogenetic analysis revealed that all sampled species in Pinaceae formed a monophyletic clade with high bootstrap value. The *Larix* is closely related to *Pseudotsuga*.

**Figure 1.**
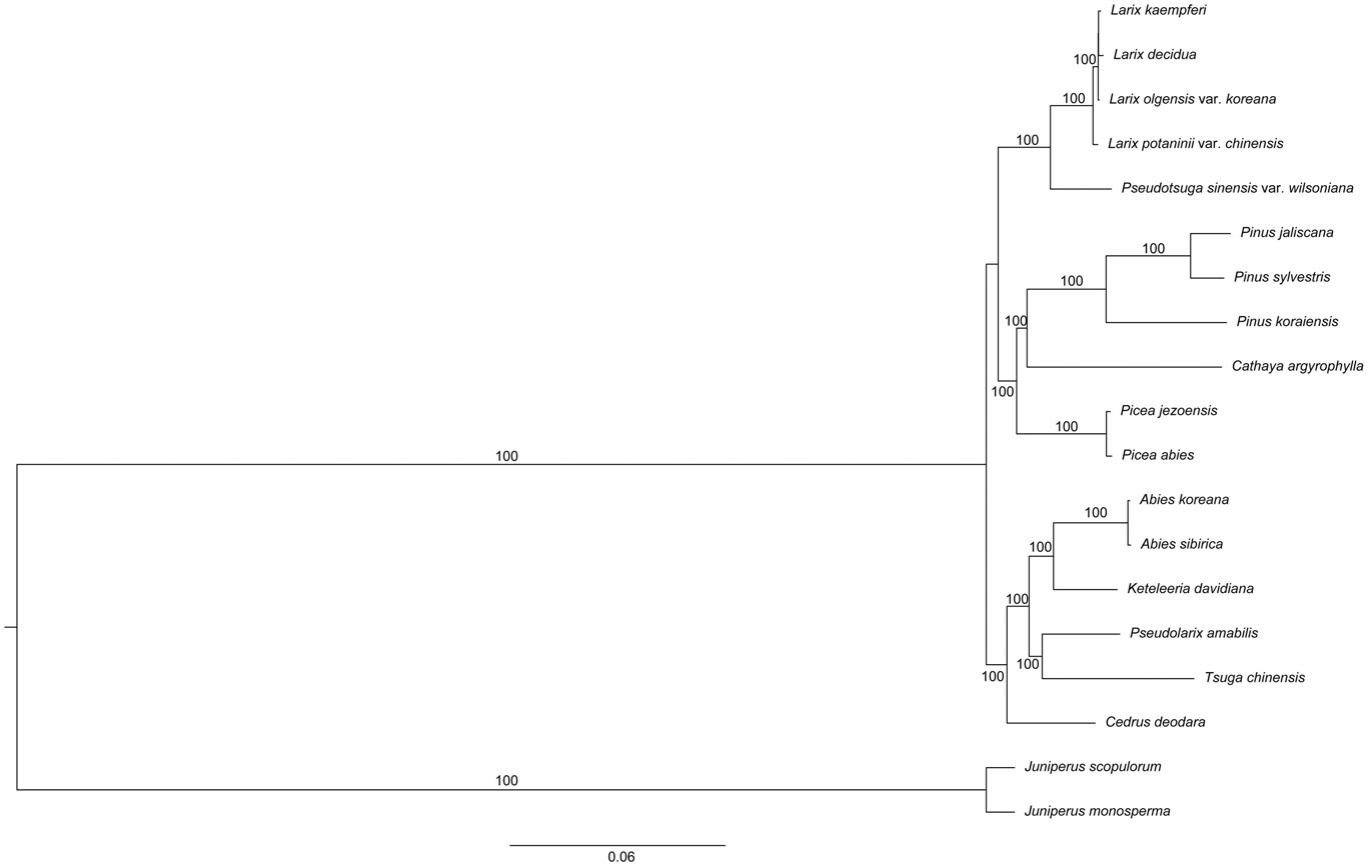
The phylogenetic tree based on the 19 complete chloroplast genome sequences. Accession Numbers: *L. kaempferi* (MF990369), *L. decidua* (NC016058), *L. olgensis* var. koreana (MF990370), *L. potaninii* var. chinensis (KX880508), *Pseudotsuga sinensis* var. wilsoniana (NC016064), *P. jaliscana* (NC035948), *P. sylvestris* (NC035069), *P. koraensis* (NC004677), *Cathaya argyrophylla* (NC014589), *Picea jezoensis* (NC029374), *P. abies* (NC021456), *Abies koreana* (NC026892), *A. sibirica* (NC035067), *Keteleeria davidiana* (NC011930), *Pseudolarix amabilis* (NC030631), *Tsuga chinensis* (NC030630), *Cedrus deodara* (NC014575), *Juniperus scopulorum* (NC024023), *J. monosperma* (NC024022).
